# Drug-Related Problems and Sick Day Management Considerations for Medications that Contribute to the Risk of Acute Kidney Injury

**DOI:** 10.3390/jcm13020343

**Published:** 2024-01-07

**Authors:** Mimi Truong, Wubshet Tesfaye, Kamal Sud, Connie Van, Shrey Seth, Nerida Croker, Ronald Lynel Castelino

**Affiliations:** 1 School of Pharmacy, Faculty of Medicine and Health, The University of Sydney, Sydney 2006, Australia; wubshet.tesfaye@sydney.edu.au (W.T.); connie.van@sydney.edu.au (C.V.); ronald.castelino@sydney.edu.au (R.L.C.); 2 Sydney Medical School, Faculty of Medicine and Health, The University of Sydney, Sydney 2006, Australia; 3 Nepean Kidney Research Centre, Department of Renal Medicine, Nepean Hospital, Nepean and Blue Mountains Local Health District, Kingswood 2747, Australia; 4 Manipal Academy of Higher Education (MAHE), The University of Sydney, Sydney 2006, Australia; 5 Meditrax, Drummoyne 2047, Australia; 6 Pharmacy Department, Blacktown Hospital, WSLHD, Blacktown 2148, Australia

**Keywords:** acute kidney injury, chronic kidney disease, sick day management, medication therapy management, medication review, descriptive study

## Abstract

Background: Medication use during acute illness increases the risk of experiencing drug related problems (DRPs), including acute kidney injuries. It is recommended that potentially nephrotoxic medications are withheld during acute illness, including sulfonylureas, angiotensin converting enzyme inhibitors, diuretics, metformin, angiotensin receptor blockers, non-steroidal anti-inflammatories and sodium glucose co-transporter 2 inhibitors (SADMANS). It is unknown if Australian pharmacists currently provide sick day medication management advice regarding SADMANS medications. Hence, we aimed to identify current DRPs and the recommendations made during residential medication management reviews (RMMRs), especially with SADMANS medications. Methods: A retrospective review of 408 RMMRs was conducted. DRPs and pharmacist recommendations were classified according to a modified DOCUMENT system. General practitioners’ (GP) recommendations were also categorised. Results: Over 97% of residents experienced at least one DRP. Common problems for non-SADMANS medications were “toxicity or adverse drug reaction”, “drug selection” and “over/underdosing” and those for SADMANS medications included “toxicity or adverse drug reaction”, “monitoring” and “drug selection”. GPs agreed with pharmacist recommendations approximately 40% of the time. No pharmacists provided sick day medication management advice for SADMANS medications. Conclusion: DRPs remain highly prevalent in aged care facilities. Medication reviews effectively identify and resolve DRPs approximately 40% of the time, but do not currently minimise the risk associated with using SADMANS medications during sick days, which is a potential area of improvement.

## 1. Introduction

People who take multiple medications, especially those who are older (>65 years) and have chronic illnesses, are at an increased risk of experiencing drug-related problems (DRPs) [1]. DRPs have been shown to contribute to approximately 2–3% of all hospital admissions and cost the Australian economy an estimated AUD $1.4 billion annually [2]. People with chronic kidney disease (CKD) are particularly vulnerable to DRPs as physiological changes related to altered kidney function impact the pharmacokinetics and pharmacodynamics of several medications [3]. Studies have reported inappropriate medication use in CKD (defined as the use of medications at higher than recommended doses or the use of contraindicated medications as per kidney function) to range between 9.4% and 81.1% [4]. Furthermore, people with CKD are also at an increased risk of adverse drug events (ADEs) during an acute illness (e.g., gastrointestinal illness with symptoms such as diarrhoea), which can lead to volume depletion, increasing the risk of developing an acute kidney injury (AKI), where there is a sudden decline in kidney function, increasing the risk of morbidity and mortality [5]. 

Specific medications potentially increasing the risk of an AKI during an acute illness include sulfonylureas, angiotensin converting enzyme inhibitors (ACEis), diuretics, metformin, angiotensin receptor blockers (ARBs), non-steroidal anti-inflammatory drugs (NSAIDs) and sodium glucose co-transporter 2 (SGLT2) inhibitors [6,7]. These medications are also referred to as SADMANS medications [6]. Currently, several organisations, including Kidney Health Australia, Diabetes Canada, National Health Service England and the United Kingdom Renal Registry, provide sick day recommendation guidelines (SDMGs), recommending that SADMANS medications should be temporarily discontinued in the event of an acute illness. However, the uptake of these recommendations remains poor, with only 15% of patients reporting having received guidance from health care professionals to withhold medications during an acute illness, and only 5% acting on such advice [6,8]. This is largely attributed to the lack of awareness and consensus among health care professionals, including GPs and pharmacists, on the definition of an acute illness and the duration of medication discontinuation [6]. Inadequate provision of SDMGs from health care professionals, therefore, potentially subjects consumers to experiencing an AKI, possibly worsening their outcomes overall [5]. 

In Australia, home medicines review (HMRs) and residential medication management review (RMMRs) are government-funded programs provided by pharmacists in collaboration with general practitioners (GPs), with the goal of supporting the quality of use of medicines by identifying, resolving and preventing DRPs [9]. Several studies have shown that RMMRs and HMRs are effective at decreasing a patient’s drug burden, decreasing the number of DRPs and improving patients’ medication knowledge and adherence, thereby improving outcomes [10,11,12]. While evidence exists on the impact of RMMRs on identifying and resolving DRPs [10,11,12], little is known about the impact of RMMRs on medications that can potentially cause more harm in people with chronic disease(s) who are acutely ill. It is also not known whether Australian pharmacists currently provide recommendations for medication management during sick days to prevent adverse drug events like AKIs from occurring. 

This study, therefore, aims to gain an overall understanding of current DRPs identified by pharmacists during RMMRs, with specific objectives to complete the following:

- Describe the most common DRPs identified by pharmacists, including medications that require sick day management (SADMANS).
- Describe the recommendations made by pharmacists to aged care staff (GPs and nurses), including recommendations on withholding medications during an acute illness.
- Describe GP uptake of pharmacist recommendations during RMMRs.

## 2. Materials and Methods

### 2.1. Data Collection, Study Population and Sampling 

This retrospective, descriptive study included an analysis of 408 de-identified RMMR reports randomly selected from one of Australia’s leading aged care medication management review providers. To be eligible for an RMMR, participants must be living in a residential aged care facility (RACF); be currently experiencing or be at risk of experiencing a medication misadventure, for instance, those who have been recently discharged or are using a medication with a narrow therapeutic index; and they must not have had a previous RMMR in the last 12 months. Hence, each RMMR represents one resident, as no repeat RMMRs were done. The RMMR service was conducted across facilities during 1–31 May 2022 by accredited pharmacists. To become accredited, pharmacists must complete a two-stage training process, then be accredited by one of the following bodies in Australia: The Society of Hospital Pharmacists Australia, The Australasian Collage of Pharmacy or The Australian Association of Consultant Pharmacy (AACP). Closure of the AACP did not occur until after these reviews were performed and AACP accreditation remained valid until 30 June 2023. 

### 2.2. Data Extraction and Coding

Participants’ demographic information, including age, sex and postcode, were extracted. Medical conditions were classified using the ICD-11 coding tool [13] and, from this, the Charlson Comorbidity Index (CCI) score was also calculated [14]. As part of calculating the CCI, where CKD stage was not specified, it was determined using the laboratory results. Where laboratory results were unavailable, CKD status could not be determined and was therefore omitted from the CCI score. When calculating the CCI score, patients were only considered as having a tumour if antineoplastic medications were taken at the time of the RMMR. Medications were classified using the Anatomic Therapeutic Chemical (ATC) classification system, which excludes complementary, homeopathic and herbal traditional medicinal products [15]. 

All DRPs identified and recommendations made by pharmacists to the GPs were categorised using an adapted version of the DOCUMENT classification system, a tool commonly used in community pharmacy to record actual and potential DRPs and clinical interventions [16,17]. Modifications of existing tools have been shown to be important in allowing for complete classification of all problems [18], which was the case where the original DOCUMENT could not adequately capture all problems and recommendations found during RMMRs. Some modifications to DOCUMENT include addition of the following categories for DRPs: (T4) cautioning against toxicity and (NC) non-clinical (Table S1). Other modifications to DOCUMENT for recommendations made by pharmacists include the following: (R3a) drug change: cease; (R3b) drug change: initiate; (R3c) drug change: cease and initiate; (R8a) drug change: combination formulation; (R9a) review prescribed medicine; (R16a) information to nursing staff; (R20) non-clinical; (R0) not classifiable (Table S2). 

In instances where the pharmacist provided multiple recommendations for one problem, the overall recommendation was determined and coded. For instance, the recommendation below was coded as (R3b) drug change: initiate rather than (R17) monitoring: laboratory test since the final goal was to initiate therapy.

‘…taking fludrocortisone and carbamazepine both may reduce bone mineral density. Consider assessing the patient’s bone mineral density if not done recently to ascertain whether they might benefit from an antiresorptive therapy.’

GP responses to pharmacist recommendations were also provided in the RMMRs and were classified as (A) accepted if they agreed, (R) rejected if they disagreed, (O) other recommendation provided if they disagreed with the pharmacist but provided an alternate solution, or (N) no response if they did not address the pharmacist’s findings.

All data were extracted by one author (M.T.) and was cross-checked with a second author (R.C.). Any discrepancies between findings were discussed between the two authors before the final code was assigned.

### 2.3. Data Analysis 

All extracted data were entered into Microsoft Excel and organised using the RStudio program. Descriptive statistics with normal distribution are presented as mean (±SD) or proportion otherwise.

## 3. Results

**Figure 1:**
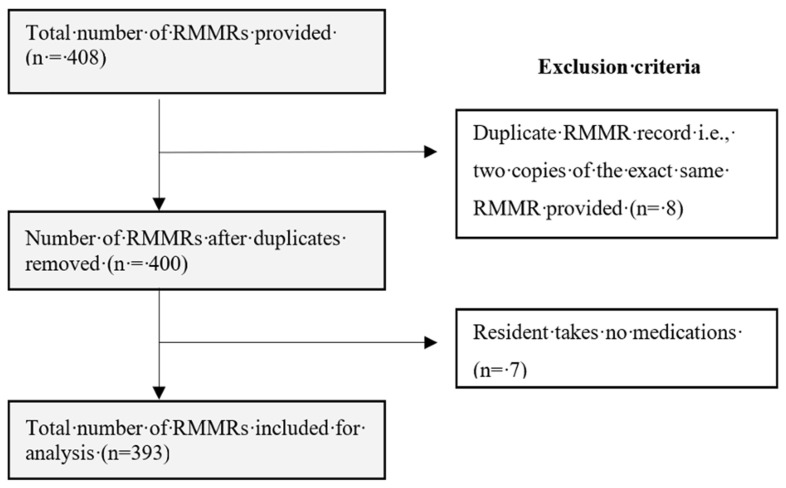
Exclusion criteria used to screen RMMRs for inclusion in analysis.

A total of 408 RMMR reports were collected from the RMMR service provider and 15 were excluded from the final analysis, as they were duplicates or the resident was not taking any medications (Figure 1). Most of the study sample (69.7%) was from major cities across Australia. The mean (SD) age of the study sample was 85.2 (8.1) years, and 63.4% of the residents were female (Table 1). The mean (SD) number of medical conditions was 8.4 (2.8), and the mean (SD) number of regular and ‘as needed’ medications taken were 9.3 (4.1) and 2.8 (2.3), respectively (Table 1).

### 3.1. Drug-Related Problems and Recommendations Found in Non-SADMANS Medications 

DRPs were highly prevalent, as 97.2% of residents experienced at least one DRP. A total of 1051 DRPs were identified by the pharmacists, where 941 (89.5%) were caused by non-SADMANS medications. For non-SADMANS medications, the most common causative medications of DRPs were those used for the nervous system (38.5%), followed by the alimentary tract and metabolism (32.0%), the cardiovascular system (11.1%), blood and blood-forming organs (5.1%) and the musculoskeletal system (4.8%) (Table 2). Although the DRPs for each medication group were unique, common problems across most medication classes related to “toxicity or ADR”, “drug selection” and “undertreated” (Table 2). A further breakdown of the types of DRPs within each category and the recommendation for the DRPs can be found in Table S3.

### 3.2. Drug-Related Problems Found in SADMANS Medications 

A total of 316 (80.2%) residents had cardiovascular disease (CVD); specifically, 211 (53.7%) patients had hypertension, 80 (20.4%) had diabetes and 53 (13.5%) had both hypertension and diabetes. CKD was documented as a diagnosis in 24 (6.1%) residents; 3 (0.8%) had early-stage CKD (stage 1–2), 20 (5.1%) had moderate CKD (stage 3–4), 1 (0.3%) had kidney failure (stage 5) and 8 (2.0%) residents had CKD, but the stage was not specified. Thirty-eight (9.7%) residents had reduced kidney function without a documented diagnosis of CKD. Six (1.5%) residents had a history of a previous AKI, three of whom had no record of reduced kidney function, two with moderate CKD and one with kidney failure. For patients with CKD, 17 (70.8%) were given at least one medication that is potentially problematic in kidney disease. Hence, 20 recommendations were made to decrease the dose of a medication cleared by the kidney, as it was inappropriate as per the resident’s kidney function, and 12 recommendations were made to cease medications as they were contraindicated as per the resident’s kidney function. 

Overall, 149 (37.9%) residents were taking at least one SADMANS medication at the time of the RMMR; 112 (28.5%) residents were taking one SADMANS medication, 33 residents were taking two SADMANS medications and 4 residents were taking more than three SADMANS medications. The highest number of SADMANS medications taken by a resident was four. SADMANS medications accounted for 191 (18.7%) of all DRPs identified by pharmacists. Diuretics were often associated with DRPs (34.0%), followed by metformin (21.5%), NSAIDs (19.4%), ARBs (11.5%), ACEis (9.4%), sulfonylureas (3.7%) and SGLT2 inhibitors (0.5%) (Table 3). Amongst all SADMANS medications, “toxicity or ADR” was a common problem, followed by “monitoring” and “drug selection” (Table 3). More detail about the types of DRPs within each category and the recommendation for the DRPs are presented in Table S4. For issues surrounding toxicity, pharmacists normally recommended monitoring, but for metformin most pharmacists recommended a dose decrease. Pharmacists were more likely to recommend dose changes or advise monitoring for patients taking SADMANS medications if they had either hypertension or diabetes. Issues around drug selection were mostly due to contraindications due to reduced kidney function (Table S4). 

Interestingly, only one pharmacist cautioned nurses and GPs on the risk of AKI occurring if the resident became dehydrated during an acute illness, but they did not provide any medication management recommendations during sick days, i.e., withhold the medication.

### 3.3. Pharmacist Recommendations and Rate of Acceptance 

A total of 997 recommendations from 393 RMMR reports were directed towards GPs and 24 were directed towards aged care staff, mostly relating to non-clinical issues. Overall, 41 (10.4%) of the RMMRs included GP responses to pharmacists’ recommendations. From the reports which included GP responses, 102 (82.9%) pertained to non-SADMANS groups of medications, while 21 (17.1%) pertained to SADMANS medications. In total, 39.2% (*n* = 40) of recommendations relating to non-SADMANS medications were accepted, while 42.9% (*n* = 9) of recommendations relating to SADMANS medications were accepted (Table 4). 

Monitoring was the most common recommendation that was widely accepted by GPs regardless of medication group (non-SADMANS/SADMANS). GPs mostly agreed to cease a medication, especially for nervous system medications. However, recommendations to increase the dose of medications or change medications were often rejected. 

## 4. Discussion

There are several key findings that have emerged from our study. Firstly, our study showed that DRPs continue to be highly prevalent in residential aged care facilities, with over 97% of residents experiencing at least one DRP. This is consistent with several local and international studies which have demonstrated that medication management for older residents in RACFs is suboptimal [18,19]. A systematic review in 2017 investigating studies conducted in the United States, the United Kingdom and Australia highlighted that medication reviews by pharmacists improved the quality of use of medicines in RACFs [20]. The findings from our study also highlight the ability of medication reviews to identify potential and actual DRPs, thus improving medication use in RACFs. Most of these improvements have been with pharmacists on a visiting basis. Hence, there have been calls for more sustainable interventions to enable system-level improvements in medication management in RACFs. Recently, the Royal Commission into Aged Care Quality and Safety found that medication management and safety is an essential area of improvement. In response, the Australian government has recently approved on-site aged care pharmacists. Findings from a pilot study showed the feasibility and acceptability of aged care pharmacists among residents, aged care staff and GPs [21], but further evidence is needed to determine if the availability of on-site pharmacists who can provide RMMRs will lead to reduced DRPs and improved clinical outcomes. 

Our study reinforced the value of pharmacist-conducted medication reviews in identifying DRPs [22], particularly in identifying issues regarding “toxicity or adverse drug reactions”, “drug selection”, “monitoring” and “over/underdosing”. This study also showed that pharmacists effectively flag medications for deprescribing [23] given that many recommendations were made to decrease the resident’s exposure to the medication. We determined that pharmacist recommendations were accepted by GPs approximately 40% of the time, which is lower than previous studies on RMMRs (over 70%) [18,19,22]. This may be explained by only a small proportion of our study including GP outcome data (10%), leading to a possible underreporting of GP acceptance. The lack of available GP data may have occurred since not all GPs provide pharmacist feedback after receiving RMMR reports. 

We observed a difference between the types of recommendations and their acceptance rate. For instance, recommendations on monitoring were generally well received, whereas changes in therapy, such as dose changes or initiating medications, were not. This is consistent with the previously reported literature [22]. Nervous system medications continue to be the biggest source of DRPs [1,18,19], often leading to pharmacists providing deprescribing recommendations such as “cease”, “cease and initiate” or “dose decrease”. This was expected given they have a significant side effect profile and are frequently prescribed for neurological conditions which are prevalent in older people [1]. The medication class that the recommendation is being made for may also influence GP acceptance, as pharmacist recommendations to cease nervous system medications were more frequently accepted than recommendations to cease cardiovascular system medications. This was also observed in other studies [24]. A possible barrier to deprescribing cardiovascular medications may be GPs requiring the prescribing specialist’s opinion beforehand [25]. This finding reinforces the idea that RMMR processes should ensure the enhanced collaboration between specialists, GPs and pharmacists in conjunction with the use of a formal deprescribing algorithm to improve the uptake of pharmacist recommendations [22,26]. A systematic review also highlighted that intense pharmacist interventions between clinicians and patients were the most successful approach to reducing polypharmacy—again highlighting the collaborative effort required to successfully deprescribe medications [27]. Reducing polypharmacy would address one of the major risk factors that contribute to ADR [1]. 

Like neurological medications, the use of SADMANS medications requires caution, given the findings from a recent study where over 75% of people hospitalised with AKI were taking at least one SADMANS medication [28]. Expert consensus has shown that SADMANS medications potentially precipitate AKI when used in patients who are acutely unwell, which is likely to be higher in older people, especially those with CVD, CKD, diabetes and those taking more than one SADMANS medication [6,7,8]. Residents from our study were potentially at risk of developing AKI during acute illness, given 25% of the residents had documented CKD/reduced kidney function and 38% of the residents were taking SADMANS medications. Specifically, 9.4% of the residents were taking more than one SADMANS medication and 1% of residents were taking more than three SADMANS medications. 

Despite the potential risk, no recommendations were made by pharmacists to GPs or aged care staff regarding sick day management guidelines (SDMGs), such as withholding SADMANS medications during an acute illness [6]. Only one RMMR recommendation identified the risk of AKI developing during an episode of acute illness, but it was for dulaglutide (a glucagon-like peptide-1 analogue), which is not an SADMANS medication. The absence of these recommendations may be due pharmacists lacking awareness about current SDMGs for patients taking SADMANS medications and poor-quality sick day resources [6,8]. The impact of poor resources was shown in a recent scoping review where only 15% of patients taking these high-risk medications were provided advice by health care professionals to withhold these medications during an acute illness, and only 5% of the patients followed this advice [6]. Furthermore, Faber et al. [29] found that in 91% of cases when patients contacted their GP with an acute illness, no sick day management advice was provided. Inadequate information provided by health care professionals explains why emergency department presentation of ADEs due to SADMANS medications remain significant [28], despite being potentially preventable. 

Barriers to pharmacists implementing SDMGs may include a lack of outcome data as guidelines are mostly based off professional consensus, a lack of knowledge and confidence, and difficulty coordinating care between GPs and pharmacists [6,8]. The latter problem is highly relevant to RMMRs given the poor response rate of GPs to pharmacist recommendations in this study (~10%). Overall, more evidence surrounding SDMGs and better collaboration between pharmacists and GPs are required to provide an enhanced RMMR service, thereby preventing ADEs and improving patient outcomes. 

### Limitations

There were some limitations to this study. Some RMMRs did not have a documented diagnosis of CKD; however, residents showed reduced renal function (via laboratory values)—this may have led to an underestimation of the burden of CKD within this cohort. There was also no indication if or when the resident had a previous RMMR and if any changes were made to their medication regimen since. This may have led to an underestimation of DRPs identified by pharmacists. The CCI scores of the cohort may have also been underestimated since solid cancer status may have been underestimated, as people with terminal cancer may no longer be taking antineoplastic agents. Furthermore, given that the RMMRs were provided by pharmacists who underwent training from the RMMR service provider, their findings and recommendations may not be generalisable to other accredited pharmacists in Australia. Another limitation is that complementary and herbal medications were excluded from this study since these medications are often administered by aged care staff and are less likely to be of significant concern than when used in community settings. However, it is known that these medications also have side effect profiles and there is some evidence on their drug interactions. Hence, these DRPs may also have been unaccounted for. Finally, the GP acceptance rate was available in only 10% of the RMMRs and it is unclear whether pharmacist recommendations were implemented or not; hence, the impact of the RMMRs could not be ascertained.

## 5. Conclusions

DRPs remain highly prevalent in residential aged care settings and RMMRs continue to be effective in identifying and resolving certain issues, like deprescribing nervous system medications. This study showed that pharmacists providing RMMRs do not currently provide advice regarding sick day medication management for people prescribed SADMANS medications, despite the risk involved with use. This suggests that further research is required to explicitly determine the knowledge gaps that pharmacists may have in this area, which will then inform further strategies to support the implementation of sick day management guidelines in the future.

## Figures and Tables

**Table 1:** Characteristics of the study sample.

Demographic Information (*n* = 393)	Value
Mean (±SD) age (years)	85.2 ± 8.1
Sex (%)	
Female	249 (63.3%)
Male	139 (35.4%)
Unidentifiable	5 (1.3%)
Remoteness (%)	
Major cities	274 (69.7%)
Regional	118 (30.0%)
Not available	1 (0.3%)
Mean (±SD) number of medical conditions	8.4 ± 2.8
Top five medical conditions [*n* (%)]	
1. Diseases of the circulatory system	630 (19.1%)
2. Mental, behavioural or neurodevelopmental disorders	532 (16.1%)
3. Diseases of the musculoskeletal system or connective tissue	382 (11.6%)
4. Endocrine, nutritional or metabolic diseases	267 (8.1%)
5. Symptoms, signs or clinical findings, not elsewhere classified	249 (7.5%)
Mean (±SD) number of regular medications	9.3 ± 4.1
Top five regular medications used [*n* (%)]	
1. Alimentary tract and metabolism	1172 (32.3%)
2. Nervous system	840 (23.2%)
3. Cardiovascular	665 (18.3%)
4. Blood and blood-forming organs	215 (5.9%)
5. Sensory organs	167 (4.6%)
Mean (±SD) number of PRN medications	2.8 ± 2.3
Top five PRN medications used [*n* (%)]	
1. Alimentary tract and metabolism	362 (33.2%)
2. Nervous system	356 (32.7%)
3. Respiratory system	107 (9.8%)
4. Dermatological	85 (7.8%)
5. Musculoskeletal	58 (5.3%)
Mean (±SD) CCI score	5.4 ± 1.7

**Table 2:** Top 5 causative medications (excluding SADMANS), problems found with these medications and most common recommendation made by pharmacists for the type of problem.

Drug Group	Proportion of DRPs (*n*%)	Types of Problems Found (*n*)	Most Common Recommendation Made by Pharmacists for the Type of Problem (*n*)
Nervous system	362 (38.5%)	Toxicity or ADR (113)	Dose decrease (27)
		Drug selection (100)	Drug change: cease (28)
		Undertreated (49)	Other changes to therapy (7)
		Over- or underdose (54)	Dose decrease (25)
		Compliance (12)	Drug formulation change (5)
		Monitoring (7)	Monitoring: laboratory test (6)
		Not classifiable (19)	Refer to prescriber (5)
		Non-clinical (8)	Non-clinical (6)
Alimentary tract and metabolism	301 (32.0%)	Drug selection (75)	Drug change: cease (18)
		Undertreated (48)	Dose increase (12)
		Toxicity or ADR (46)	Dose decrease (12)
		Over- or underdose (56)	Dose decrease (24)
		Monitoring (25)	Monitoring: laboratory test (24)
		Compliance (24)	Drug formulation change (11)
		Not classifiable (14)	Drug change: cease (6)
		Non-clinical (13)	Information to nursing staff (7)
Cardiovascular system	104 (11.1%)	Toxicity or ADR (47)	Monitoring: laboratory test (8) Drug change: cease (4) Dose decrease (1)
		Over- or underdose (17)	Dose decrease (7)
		Drug selection (19)	Drug change: cease and initiate (6)
		Monitoring (11)	Monitoring: laboratory test (8)
		Not classifiable (4)	Monitoring: non-laboratory test (1) Dose frequency/schedule change (1) Other changes to therapy (1) Review prescribed medicine (1)
		Undertreated (4)	Drug change: initiate (3)
		Compliance (2)	Dose frequency/schedule change (1) Other change to therapy (1)
Blood and blood-forming organs	48 (5.1%)	Toxicity or ADR (17)	Monitoring: laboratory test (10)
		Drug selection (10)	Drug change: cease and initiate (4)
		Over- or underdose (9)	Review prescribed medicine (2) Dose increase (2)
		Monitoring (5)	Monitoring: laboratory test (4)
		Undertreated (5)	Drug change: initiate (4)
		Compliance (1)	Information to nursing staff (1)
		Non-clinical (1)	Review prescribed medicine (1)
Musculoskeletal system	45 (4.8%)	Undertreated (19)	Drug change: initiate (11)
		Drug selection (8)	Review prescribed medicine (4)
		Toxicity or ADR (5)	Monitoring: laboratory test (3)
		Monitoring (3)	Monitoring: laboratory test (3)
		Over- or underdose (3)	Dose increase (1) Review prescribed medicine (1) Refer to prescriber (1)
		Not classifiable (3)	Monitoring: laboratory test (2)
		Compliance (2)	Refer to prescriber (1) Education/counselling session (1)
		Non-clinical (2)	Non-clinical (2)

**Table 3:** Problems found with SADMANS medications and most common recommendation made by pharmacists for the type of problem.

Drug Group	Proportion of DRPs (*n*%)	Types of Problems Found (*n*)	Most Common Recommendation for the Type of Problem (*n*)
Sulfonylureas	7 (3.7%)	Drug selection (4)	Drug change: cease and initiate (2) Drug formulation change (2)
		Toxicity (2)	Drug change: cease and initiate (1) Review prescribed medicine (1)
		Over- or underdose (1)	Monitoring: laboratory test (1)
ACEis	18 (9.4%)	Toxicity (9)	Monitoring: laboratory test (5)
		Monitoring (3)	Monitoring: laboratory test (2)
		Not classifiable (2)	Dose decrease (1) Monitoring: non-laboratory test (1)
		Over- or underdose (2)	Dose decrease (2)
		Drug selection (1)	Drug change: cease and initiate (1)
		Undertreated (1)	Drug change: initiate (1)
Diuretics	65 (34.0%)	Toxicity (32)	Monitoring: laboratory test (21)
		Monitoring (10)	Monitoring: laboratory test (9)
		Drug selection (10)	Review prescribed medicine (4)
		Over- or underdose (7)	Dose decrease (2) Non-clinical (2)
		Not classifiable (2)	Dose decrease (1) Review prescribed medicine (1)
		Undertreated (3)	Monitoring: non-laboratory test (1) Dose decrease (1) Drug change: initiate (1)
		Compliance (1)	Review prescribed medicine (1)
Metformin	41 (21.5%)	Monitoring (12)	Monitoring: laboratory test (9)
		Toxicity (10)	Dose decrease (6)
		Drug selection (8)	Drug change: combination formulation (4)
		Over- or underdose (7)	Dose decrease (3)
		Undertreated (3)	Dose decrease (1) Drug change: initiate (1) Monitoring: laboratory test (1)
		Not classifiable (1)	Monitoring: laboratory test (1)
ARBs	22 (11.5%)	Toxicity (12)	Monitoring: non-laboratory test (5)
		Monitoring (5)	Monitoring: laboratory test (3)
		Undertreated (2)	Monitoring: non-laboratory test (1) Review prescribed medicine (1)
		Drug selection (1)	Dose decrease (1)
		Over- or underdose (1)	Drug chance: cease (1)
		Non-clinical (1)	Non-clinical (1)
NSAIDs	37 (19.4%)	Toxicity (17)	Monitoring: laboratory test (9)
		Drug selection (10)	Review prescribed medicine (4)
		Undertreated (4)	Drug change: initiate (4)
		Monitoring (2)	Monitoring: laboratory test (1)
		Non-clinical (2)	Non-clinical (2)
		Over- or underdose (2)	Dose decrease (1) Review prescribed medicine (1)
SGLT2 inhibitors	1 (0.5%)	Undertreated (1)	Monitoring: laboratory test (1)

**Table 4:** GP acceptance of pharmacist recommendations.

Pharmacist Recommendation	Medication Group (Non-SADMANS/SADMANS)	Recommendation Accepted	Other Recommendation Provided	Recommendation Rejected	No Response to Recommendation
Dose decrease	Non-SADMANS	3	4	2	1
	SADMANS	1	2	0	0
Dose increase	Non-SADMANS	1	0	2	4
	SADMANS	0	1	1	0
Drug change: initiate	Non-SADMANS	3	2	1	1
	SADMANS	0	1	0	0
Dose frequency/schedule change	Non-SADMANS	0	1	0	4
	SADMANS	0	0	0	1
Review prescribed medicine	Non-SADMANS	6	5	2	1
	SADMANS	0	1	0	0
Monitoring: laboratory test	Non-SADMANS	9	0	0	2
	SADMANS	5	0	0	2
Monitoring: non-laboratory test	Non-SADMANS	2	1	0	2
	SADMANS	2	0	0	1
Drug change	Non-SADMANS	1	0	0	0
Drug change: cease		4	3	2	3
Drug change: cease and initiate		6	3	4	2
Drug formulation change		1	0	0	0
Refer to prescriber		3	2	1	2
Other referral required		0	0	0	1
Education/counselling session		0	1	0	0
Information to nursing staff		1	0	0	1
Not classifiable		0	1	1	0
Other changes to therapy	SADMANS	0	0	0	1
Non-clinical		1	0	0	1
Total		49	28	16	30

## Data Availability

Restrictions apply to the availability of these data.

## Supplementary Materials

The following supporting information can be downloaded at: https://www.mdpi.com/article/10.3390/jcm13020343/s1, Table S1: Modified DOCUMENT DRPs and examples from RMMRs; Table S2: Modified DOCUMENT recommendations and examples from RMMRs; Table S3: Full description of drug-related problems and recommendations made for non-SADMANS medications; Table S4: Full description of drug-related problems and recommendations made for SADMANS medications.
