# Supplementary material for: Drug-Related Problems and Sick Day Management Considerations for Medications that Contribute to the Risk of Acute Kidney Injury
Source: J Clin Med. 2024 Jan 7;13(2):343. doi: 10.3390/jcm13020343 (PMC10816903; doi:10.3390/jcm13020343)
Supplement: Supplementary file 1 [file jcm-13-00343-s001.zip › jcm-2769610-supplementary.pdf]

Supplementary material

Table S1: Modified DOCUMENT DRPs and examples from RMMRs

Code	Drug related problem	Description	Example
D1	Duplication	Inappropriate use of two drugs from the same therapeutic class	"Mrs is administered 2 beta blockers; metoprolol and propranolol. Consider reviewing benefits versus risk..."
D2	Drug interaction	Likely interaction between two prescribed drugs (no symptoms evident yet)	"Pantoprazole in the morning may reduce absorption of thyroxine"
D3	Wrong Drug	Incorrect drug supplied, either by an incorrect doctor's prescription or incorrectly dispensed by the pharmacy	"…she was commenced on citalopram…currently administered escitalopram"
D4	Incorrect strength	Incorrect or no details about strength of medication supplied	N/A
D5	Inappropriate dosage form	Formulation is inappropriate in terms of the intended use of the product	"In place of current tablets that are crushed, consider liquid formulation like OsteVit-D that can be administered once a week"
D6	Contraindications apparent	Patient has a contraindication or precaution to the drug being used due to their medical conditions, or a previous allergy to the drug or drug group	"Hiprex is ineffective in renal impairment because of inadequate concentrations in renal tubules. In view of his renal impairment, consider a review of the ongoing benefit of Hiprex tablets"
D7	No indication apparent	No clear indication for the use of the drug	"PRN Hydrozole appears charged post-groin excoriation event… he does not require use of Hydrozole"
D0	Other drug selection problem	Other drug selection issues, such as the patient is taking expired medication or a more effective drug than the one prescribed is available	N/A
O1	Prescribed dose too high	Total daily dose exceeds guidelines, either due to reference dose ranges or patient parameters (age, renal function etc.)	"Mrs X is administered metformin 1g twice

			daily…The maximum recommended metformin dose is 1g daily if CrCl is between 30 and 60 mL/min”
O2	Prescribed dose too low	Total daily dose is not adequate for treatment	“Mrs X is charted for a low dose of allopurinol which may not be therapeutic… it would be worth reviewing the ongoing need for allopurinol”
O3	Incorrect/unclear dosing instructions	Specified dosing frequency/schedule or duration of treatment is unclear or incorrect	“Suggest changing the duration of application for clotrimazole cream…to minimise the risk of antimicrobial resistance”
O0	Other dose problem	Other dose related problem, such as incorrect frequency or schedule	N/A
C1	Taking too little	Patient using too little of the medication due to forgetfulness or poor understanding of therapy	“Her signing sheet suggests that she refuses her dinner time paracetamol dosage”
C2	Taking too much	Patient using too much of the medication due to forgetfulness or poor understanding of therapy	N/A
C3	Erratic use of medication	Patient using medication on an erratic basis	“I noted she refused diclofenac gel 4 times last week. Please ensure compliance…”
C4	Intentional drug misuse (including OTC medications)	Suspected overuse of a drug that is potentially abused	N/A
C5	Difficulty using dosage form	Patient has a physical problem using the dosage form due to swallowing difficulties, manual dexterity etc.	“She may not have the inspiratory effect to inhale the dose from the Turbuhaler device”
C0	Other compliance problem	Other compliance issues, such as patient choosing not to take the medication due to the product information or a media release etc.	“…PRN analgesic was given for behaviour without evidence of pain, so it is very likely to be considered as restraint.”
U1	Condition undertreated	Patient has a symptom or condition that is not currently being treated adequately	“Consider if Mr X may benefit from low dose Norspan to assist with pain and comfort should he have chronic pain associated with his legs”

U2	Condition untreated	Patient has a symptom or condition that is not being treated	"Due to institutionalised nature, she may develop deficiency of vitamin D"
U3	Preventative therapy required	Patient requires additional therapy to prevent an adverse event occurring (due to patient's therapy, coexisting diseases or risk factors)	"Given the increased risk of bleeding, consider a low dose PPI as gastroprotection…"
U0	Other untreated problem	Other untreated indication problem	N/A
M1	Laboratory monitoring	Patient requires a laboratory test, such as serum electrolyte or drug levels (no symptoms evident yet)	"…I was not able to locate any blood test report. Query if rechecking UEC and magnesium may be desirable…"
M2	Non laboratory monitoring	Patient requires a non-laboratory test, such as BP, BSL or weight check (no symptoms evident yet)	"Consider checking BGL's post meals…"
M0	Other monitoring problem	Other monitoring problem, such as patient unable to afford monitoring	N/A
E1	Patient requests drug information	Patient requests information about their medication	N/A
E2	Patients requests disease management advice	Patient requests information about the management or prevention of a condition	N/A
E3	Confusion about therapy or condition	Patient has a poor understanding of their medical condition, but their medication compliance appears to be adequate according to the dispensing history	N/A
E4	Demonstration of device	Patient has a technical problem with the administration of a device	N/A
E0	Other education or information problem	Other education problem, such as another health professional requests information	N/A
N0	Clinical interventions that cannot be classified under any other category	Clinical interventions that the pharmacist feels does not belong elsewhere (must still be a clinical problem, not administrative)	N/A
T1	Toxicity caused by dose	Patient has signs or symptoms of an adverse reaction that is likely to be dose-related	N/A
T2	Toxicity caused by drug interaction	Patient has signs or symptoms of an adverse reaction that is likely to be related to the presence of an interacting drug	N/A
T3	Toxicity evident	Patient experiencing symptoms of toxicity where there is a suspected medication cause	N/A
T4 **	Cautioning against toxicity	Patient at risk of toxicity/adverse drug reaction but no symptoms yet. Pharmacists may warn of side effects/cautions to take when using the medication e.g. risk of falls, sedation etc.	"…to minimise risk of anticholinergic effects, confusion, dependence, and tolerance, particularly

			as risks may be increased for her advanced age…”
T0	Other toxicity evident	Other toxicity suspected of being related to a drug	N/A
NC **	Non-clinical (NC)	Non-clinical issues, often documentation/administrative discrepancies.	“…indication for venlafaxine (depression)…were written by the doctor. These were not shown on the electronic medication profile”

**New categories added

Table S2: Modified DOCUMENT recommendations and examples from RMMR

Code	Recommendation	Description	Example from RMMR
R1	Dose decrease	Pharmacist recommends the daily dose of medication is decreased	“…suggest a dose reduction to 1 tablet…”
R2	Dose increase	Pharmacist recommends the daily dose of medication is increased	“…slight increase in aperient may be considered…”
R3	Drug change	Pharmacist recommends a change in current medications, such as initiating or ceasing a medication	“...remove later in the evening so that the nitrate free period is [smaller]”
R3a **	Drug change: cease	Pharmacist specifies ceasing a medication	“…consider ongoing need for medication with view to cease for now”
R3b **	Drug change: initiate	Pharmacist specifies initiating a medication	“You may wish to consider addition of regular paracetamol to reduce dependence on opioid analgesic”
R3c **	Drug change: cease and initiate	Pharmacist specifies ceasing a medication and initiating another	“…trialling a switch to a different antidepressant such as fluoxetine…”
R4	Drug formulation change	Pharmacist recommends a change in formulation that does not alter the drug or its total daily dose	“…consider switching to immediate release formulation”
R5	Drug brand change	Pharmacist recommends a change in the brand to improve compliance or due to stock unavailability etc	N/A
R6	Dose frequency/schedule change	Pharmacist suggests a change in the number of times per day or timing of the doses, without changing the total daily dose	“It has an onset effect of 6-12 hour. Review this timeslot if appropriate”
R7	Prescription not dispensed	Pharmacist does not dispense the prescription due to the circumstances, such as when the patient needs to visit the prescriber prior to dispensing	N/A

R8	Other changes to therapy	Pharmacist recommends another change to patient's current therapy	"…query if a trial of topical NSAID may be considered before taking PRN celecoxib"
R8a **	Drug change: combination formulation	Pharmacist recommends to changing an individual drug formulation to a combined drug formulation.	"Suggest combining ezetimibe and rosuvastatin (i.e. Rosuzet) to reduce pill burden"
R9	Refer to prescriber; prescriber to continue monitoring, review, etc…	Pharmacist refers patient to their prescriber to resolve the DRP	"Consider assessment of depression symptoms for e.g. with a Cornell score…"
R9a **	Review prescribed medicine	Pharmacist recommends to specifically review the current prescribed medications	"A review of his antihypertensive regime may be warranted"
R10	Refer to hospital	Pharmacist refers patient to the hospital to resolve the DRP	N/A
R11	Refer for medication review	Pharmacist recommends patient have a medication review to resolve the DRP (known as a Home Medications Review or HMR in Australia where a pharmacist visits the patient at home and sends a clinical review letter to their treating physician)	N/A
R12	Other referral required	Pharmacist refers patient to another health professional to resolve the DRP, such as a dentist, podiatrist, diabetes educator etc	"Mr X may benefit from assessment by the geriatrician as well as possibly the psychiatric team…"
R13	Education/counselling session	Pharmacist provides a detailed counselling or education session to the patient to resolve the DRP	"… she was encouraged to report worsening bowel issue…"
R14	Written summary of medications	Pharmacist provides patient with a detailed list of their medications to resolve the DRP	N/A
R15	Commence dose administration aid	Pharmacist suggests that the patient start using a dose administration aid (such as a Webster pack or dosette box)	N/A
R16	Other written information	Pharmacist provides other written information, such as Self Care cards	N/A
R16a **	Information to nursing staff	Pharmacist provides information to nursing staff to resolve DRP	"Staff need to monitory Mr X for signs and symptoms indicative of a bleed"
R17	Monitoring: laboratory test	Pharmacist suggests that the prescriber undertake some laboratory monitoring to monitor for DRPs	"...advise checking her current Ca…before each 6-monthly dose"

R18	Monitoring: non-laboratory test	Pharmacist suggests that the patient or prescriber undertake some non-laboratory monitoring to monitor for DRPs	"…monitor BGL at bedtime for 1 week…"
R19	No recommendation necessary	Pharmacist has investigated the problem and finds that the problem does not need to be addressed with any changes	"Given the pregabalin dose is being withdrawn, this may resolve the current issue"
R20 **	Non-clinical e.g. update document	Pharmacists makes a non-clinical recommendation to resolve issue.	"Suggest updating his medication list as appropriate"
R0 **	Not classifiable	Pharmacist recommendation cannot be classified by any other category	"Reconciliation of allergy information would be suggested for uniformity across the system"

**New categories added

Table S3: Full description of drug-related problems and recommendations made for non-SADMANS medications

**Drug Group**	**Proportion of DRP (n%)**	**Type of problems found (n)**	**Most common recommendation made by pharmacists for the type of problem (n)**
Nervous system	362 (38.5%)	Cautioning against toxicity (79)	Dose decrease (15)
		No indication apparent (58)	Drug change: cease (24)
		Condition undertreated (43)	Other changes to therapy (7)
		Toxicity caused by dose (28)	Dose decrease (11)
		Prescribed dose too high (24)	Dose decrease (19)
		Other drug selection problem (20)	Drug change: cease and initiate (10)
		Not classifiable (19)	Refer to prescriber (5)
		Other dose problem (16)	Dose decrease (6)
		Contraindications apparent (13)	Dose decrease (4)
		Incorrect/unclear dosing instructions (13)	Non-clinical (6)
		Non-clinical (8)	Non-clinical (6)
		Laboratory monitoring (7)	Monitoring: laboratory test (6)
		Difficulty using dosage form (6)	Drug formulation change (4)
		Inappropriate dosage form (5)	Drug formulation change (4)
		Toxicity evident (5)	Drug change: cease and initiate (2)
		Preventative therapy required (3)	Drug change: initiate (2)
		Erratic use of medication (2)	Dose increase (1)<br>Drug formulation change (1)
		Duplication (2)	Drug change: cease (2)
		Prescribed dose too low (2)	Dose increase (1)<br>Drug formulation change (1)

		Condition untreated (2)	Other referral required (1)<br>Review prescribed medicine (1)
		Other compliance problem (2)	Refer to prescriber (2)
		Taking too little (1)	Dose decrease (1)
		Drug interaction (1)	Other changes to therapy (1)
		Wrong drug (1)	Review prescribed medicine (1)
		Other toxicity problem (1)	Dose decrease (1)
		Other undertreated problem (1)	Review prescribed medicine (1)
Alimentary tract and metabolism	301 (32.0%)	No indication apparent (40)	Drug change: cease (17)
		Condition undertreated (38)	Dose increase (12)
		Cautioning against toxicity (35)	Dose decrease (12)
		Laboratory monitoring (24)	Monitoring: laboratory test (24)
		Other dose problem (23)	Dose decrease (15)
		Other drug selection problem (17)	Drug change: cease and initiate (10)
		Incorrect/unclear dosing instructions (16)	Dose frequency/schedule change (6)
		Difficulty using dosage form (15)	Drug formulation change (11)
		Not classifiable (14)	Drug change: cease (6)
		Non-clinical (13)	Information to nursing staff (7)
		Prescribed dose too high (13)	Dose decrease (9)
		Toxicity caused by dose (11)	Drug change: cease (4)
		Contraindications apparent (8)	Dose decrease (2)
		Inappropriate dosage form (7)	Drug formulation change (7)
		Erratic use of medications (6)	Drug change: cease (2)
		Condition untreated (5)	Dose increase (2)<br>Drug change: initiate (2)
		Preventative therapy required (5)	Drug change: initiate (3)
		Prescribed dose too low (4)	Dose increase (4)
		Taking too little (2)	Drug formulation change (2)
		Duplication (2)	Drug change: cease (1)<br>Drug change: cease and initiate (1)
		Other compliance problem (1)	Drug change: cease (1)
		Drug interaction (1)	Other referral required (1)
		Non laboratory monitoring (1)	Monitoring: non laboratory test (1)
Cardiovascular system	104 (11.1%)	Cautioning against toxicity (31)	Monitoring: laboratory test (7)
		Toxicity caused by dose (14)	Drug change: cease (4)
		Other dose problem (11)	Dose decrease (4)
		Other drug selection problem (9)	Drug change: cease and initiate (5)
		Laboratory monitoring (9)	Monitoring: laboratory test (8)
		No indication apparent (5)	Drug change: cease (2)<br>Review prescribed medicine (2)
		Contraindications apparent (4)	Monitoring: laboratory test (1)

			Drug change: cease (1)<br>Drug change: cease and initiate (1)<br>Dose frequency/schedule change (1)
		Not classifiable (4)	Monitoring: non laboratory test (1)<br>Dose frequency/schedule change (1)<br>Other changes to therapy (1)<br>Review prescribed medicine (1)
		Condition undertreated (4)	Drug change: initiate (3)
		Prescribed dose too high (3)	Dose decrease (3)
		Prescribed dose too low (2)	Dose increase (2)
		Non laboratory monitoring (2)	Monitoring: non laboratory test (2)
		Toxicity evident (2)	Dose decrease (1)<br>Monitoring: laboratory test (1)
		Taking too little (1)	Dose frequency/schedule change (1)
		Erratic use of medications (1)	Other change to therapy (1)
		Duplication (1)	Review prescribed medicine (1)
		Incorrect/unclear dosing instructions (1)	Non-clinical (1)
Blood and blood forming organs	48 (5.1%)	Cautioning against toxicity (14)	Monitoring: laboratory test (8)
		Contraindications apparent (4)	Drug change: cease and initiate (2)<br>Monitoring: laboratory test (2)
		Laboratory monitoring (4)	Monitoring: laboratory test (4)
		Incorrect/unclear dosing instructions (4)	Review prescribed medicine (2)
		Toxicity caused by dose (3)	Monitoring: laboratory test (2)
		Preventative therapy required (3)	Drug change: initiate (2)
		Other drug selection problem (2)	Drug change: cease and initiate (2)
		No indication apparent (2)	No recommendation necessary (1)<br>Review prescribed medicine (1)
		Prescribed dose too high (2)	Dose decrease (1)<br>Monitoring: laboratory test (1)
		Prescribed dose too low (2)	Dose increase (2)
		Condition undertreated (2)	Drug change: initiate (2)
		Other compliance problem (1)	Information to nursing staff (1)
		Drug interaction (2)	Dose frequency/schedule change (2)
		Non laboratory monitoring (1)	Monitoring: non laboratory test (1)

		Non-clinical (1)	Review prescribed medicine (1)
		Other dose problem (1)	Drug change: cease (1)
Musculoskeletal system	45 (4.8%)	Preventative therapy required (16)	Drug change: initiate (10)
		No indication apparent (7)	Review prescribed medicine (4)
		Cautioning against toxicity (5)	Monitoring: laboratory test (3)
		Laboratory monitoring (3)	Monitoring: laboratory test (3)
		Not classifiable (3)	Monitoring: laboratory test (2)
		Non-clinical (2)	Non-clinical (2)
		Condition untreated (2)	Drug change: initiate (1)<br>Monitoring: laboratory test (1)
		Other compliance problem (1)	Refer to prescriber (1)
		Erratic use of medication (1)	Education/counselling session (1)
		Contraindications apparent (1)	Monitoring: laboratory test (1)
		Other dose problem (1)	Dose increase (1)
		Prescribed dose too low (1)	Review prescribed medicine (1)
		Incorrect/unclear dosing instructions (1)	Refer to prescriber (1)
		Condition undertreated (1)	Information to nursing staff (1)

Table S4: Full description of drug-related problems and recommendations made for SADMANS medications.

**Drug Group**	**Proportion of DRP (n%)**	**Type of problems found (n)**	**Most common recommendation for the type of problem (n)**
Sulfonylurea	7 (3.7%)	Other drug selection problem (2)	Drug change: cease and initiate (2)
		Inappropriate dosage form (2)	Drug formulation change (2)
		Cautioning against toxicity (2)	Drug change: cease and initiate (1)<br>Review prescribed medicine (1)
		Other dose problem (1)	Monitoring: laboratory test (1)
ACEi	18 (8.9%)	Cautioning against toxicity (6)	Monitoring: laboratory test (5)
		Toxicity caused by dose (3)	Drug chance: cease (2)
		Laboratory monitoring (2)	Monitoring: laboratory test (2)
		Not classifiable (2)	Dose decrease (1)<br>Monitoring: non laboratory test (1)
		Other dose problem (2)	Dose decrease (2)
		Duplication (1)	Drug change: cease and initiate (1)
		Non-laboratory monitoring (1)	Monitoring: non laboratory test (1)
		Preventative therapy required (1)	Drug change: initiate (1)
Diuretics	65 (34.0%)	Cautioning against toxicity (28)	Monitoring: laboratory test (19)
		Laboratory monitoring (9)	Monitoring: laboratory test (9)
		No indication apparent (7)	Review prescribed medicine (4)

		Toxicity caused by dose (4)	Monitoring: laboratory test (2)
		Other drug selection problem (3)	Drug change: cease and initiate (2)
		Other dose problem (3)	Monitoring: laboratory test (1)<br>Dose decrease (1)<br>Dose increase (1)
		Incorrect/unclear dosing instructions (3)	Non-clinical (2)
		Not classifiable (2)	Dose decrease (1)<br>Review prescribed medicine (1)
		Condition undertreated (2)	Monitoring: non laboratory test (1)<br>Dose decrease (1)
		Erratic use of medication (1)	Review prescribed medicine (1)
		Non-laboratory monitoring (1)	Monitoring: non laboratory test (1)
		Prescribed dose too high (1)	Dose decrease (1)
		Preventative therapy required (1)	Drug change: initiate (1)
Metformin	41 (21.5%)	Laboratory monitoring (9)	Monitoring: laboratory test (9)
		Cautioning against toxicity (7)	Dose decrease (3)
		Other drug selection problem (5)	Drug change: combination formulation (4)
		Other dose problem (4)	Dose decrease (1)<br>Dose increase (1)<br>Monitoring: laboratory test (1)<br>Review prescribed medicine (1)
		Non-laboratory monitoring (3)	Monitoring: non laboratory test (3)
		Prescribed dose too high (3)	Dose decrease (2)
		Inappropriate dosage form (2)	Drug formulation change (2)
		Toxicity caused by dose (2)	Dose decrease (2)
		Condition undertreated (2)	Dose decrease (1)<br>Drug change: initiate (1)
		No indication apparent (1)	Drug chance: cease (1)
		Not classifiable (1)	Monitoring: laboratory test (1)
		Toxicity evident (1)	Dose decrease (1)
		Preventative therapy required (1)	Monitoring: laboratory test (1)
ARB	22 (11.5%)	Cautioning against toxicity (8)	Monitoring: non laboratory test (4)
		Laboratory monitoring (3)	Monitoring: laboratory test (3)
		Toxicity caused by dose (3)	Dose decrease (1)<br>Monitoring: non laboratory test (1)<br>Review prescribed medicine (1)
		Non laboratory monitoring (2)	Monitoring: non laboratory test (2)
		Condition undertreated (2)	Monitoring: non laboratory test (1)

			Review prescribed medicine (1)
		Contraindication apparent (1)	Dose decrease (1)
		Other dose problem (1)	Drug chance: cease (1)
		Non-clinical (1)	Non-clinical (1)
		Toxicity caused by drug interaction (1)	Drug chance: cease (1)
NSAIDs	37 (18.3%)	Cautioning against toxicity (15)	Monitoring: laboratory test (9)
		No indication apparent (7)	Review prescribed medicine (4)
		Preventative therapy required (4)	Drug change: initiate (4)
		Contraindication apparent (2)	Drug chance: cease (2)
		Laboratory monitoring (2)	Monitoring: laboratory test (1)
		Non-clinical (2)	Non-clinical (2)
		Prescribed dose too high (2)	Dose decrease (1)<br>Review prescribed medicine (1)
		Other drug selection problem (1)	Drug change: cease and initiate (1)
		Toxicity caused by dose (1)	Drug chance: cease (1)
		Toxicity evident (1)	Review prescribed medicine (1)
SGLT2 inhibitors	1 (0.5%)	Condition undertreated (1)	Monitoring: laboratory test (1)
